# Supplementary figures and images for: Nitric Oxide Production and Effects in Group B Streptococcus Chorioamnionitis
Source: Pathogens. 2022 Sep 28;11(10):1115. doi: 10.3390/pathogens11101115 (PMC9608865; doi:10.3390/pathogens11101115)

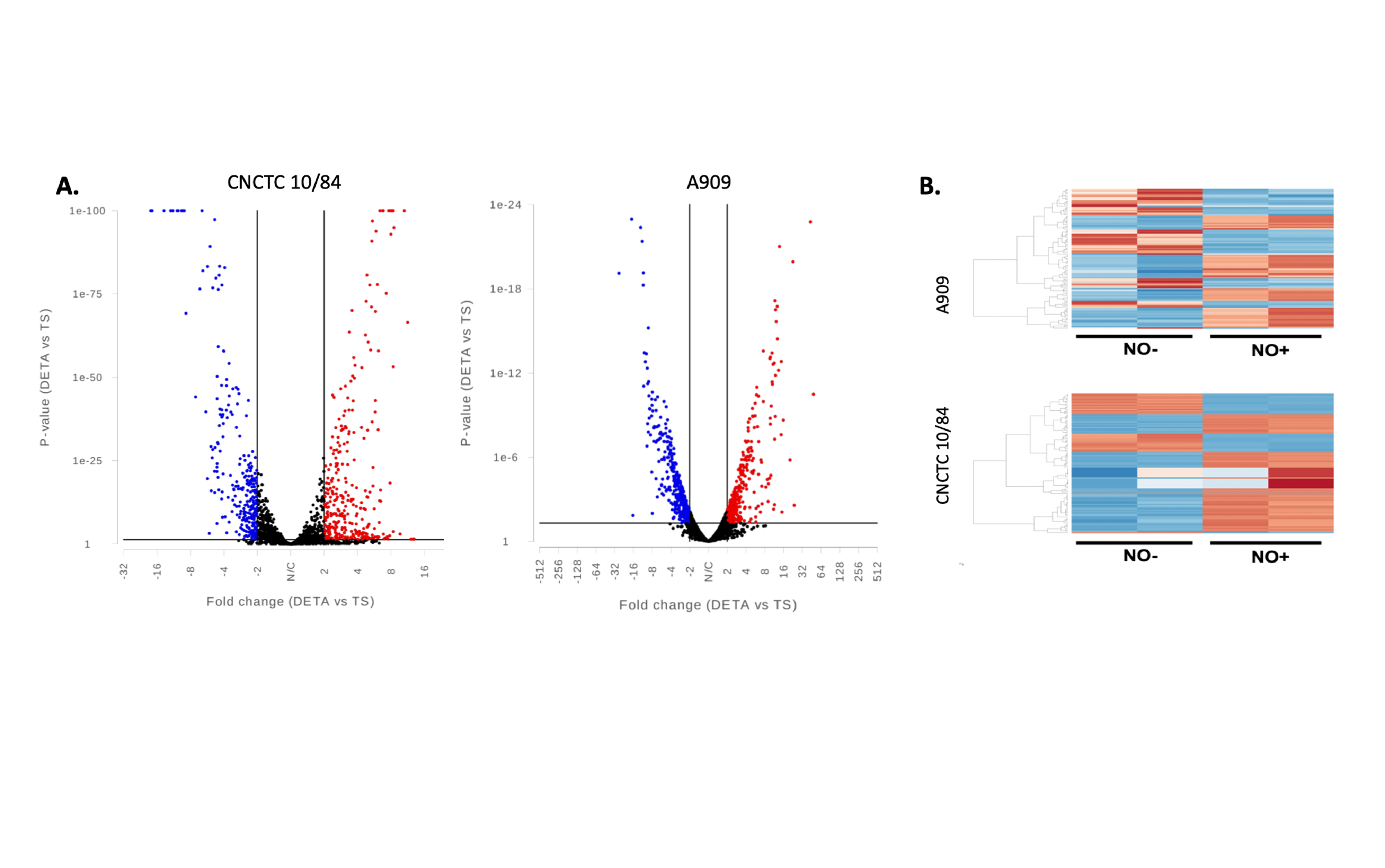

Supplement: Supplementary file 1 [file pathogens-11-01115-s001.zip › pathogens-1910809-supplementary Figure S1.tiff]
